# Real‐world antiseizure medication treatment outcomes in drug‐resistant focal epilepsy patients

**DOI:** 10.1002/epi4.12845

**Published:** 2023-10-31

**Authors:** Hind T. Hatoum, Steve Arcona, Jianbin Mao, Surrey Walton

**Affiliations:** ^1^ Pharmacy Systems Outcomes and Policy University of Illinois Chicago Chicago Illinois USA; ^2^ Global Value & Access, Cerevel Therapeutics Cambridge Massachusetts USA

**Keywords:** anti‐seizure medications, drug resistance, focal epilepsy, real‐world evidence, risk characteristics

## Abstract

**Objective:**

To gather real‐world evidence on antiseizure medications (ASMs) treatment patterns and related outcomes in patients with drug‐resistant focal epilepsy.

**Methods:**

Medical insurance claims from the start of 2014 till the end of 2019 were used. Patient selection criteria included International Classification of Diseases (ICD) codes followed by documented ASM use. Baseline patient demographics along with ASM and rescue medication use patterns and related patient outcome were documented for first (index) ASM regimen. Patients who failed the first regimen and then failed the second regimen were considered drug resistant. Multivariate analyses were performed to identify risks and other characteristics for positive or negative treatment outcomes.

**Results:**

Study cohort consisted of 46 474 patients with a mean age of 47.23 (SD: 16.94). Levetiracetam was the most first‐encountered ASM (37.94%). At baseline, 87.14% were treated with ASMs prior to having study‐confirmed diagnoses. Mental comorbidities were present in 37.86% of patients. After first‐year ASM treatment, 34.61% of patients persisted on their index regimen and 5.91% were seizure‐free. Patients failing first ASM regimen numbered 12 868 (27.69%). Drug‐resistant patients who failed first and then second ASM regimens numbered 6335 (49.23%). Percentages of patients who had successful second treatment and seizure‐free were 21.32 and 3.65, respectively.

Initiating patients on lamotrigine or carbamazepine (relative to levetiracetam), baseline use of index ASM, rescue medications, and older age or male gender all lowered the risk for treatment failure. Having higher comorbidity, comorbid mental illness, headache, or neoplasty increased such a risk. Baseline use of index ASM, depressive episode, or anxiety disorder all entailed higher risk of failing second ASM treatment.

**Significance:**

Overall, reported findings indicated that patient history at baseline and the early selection of an ASM all influenced treatment outcomes. Findings pointed to the complex nature of ASM treatment in drug‐resistant focal epilepsy patients calling for additional research to identify the optimal treatment to achieve beneficial patient outcomes.

**Key Points**

- Antiseizure medication outcome was examined in drug‐resistant epilepsy patients using claims data
- Most patients were drug experienced at baseline, over 1/3 of patients persisted on their first‐drug regimen at 1 year posttreatment
- 49% of patients who failed the first regimen failed their second regimen and were then considered as drug resistant
- Comorbidities increased the risk of failure while older age and male gender lowered the risk
- Mental illnesses present in over 1/3 of the study cohort increased risk for treatment failure

## 1 INTRODUCTION

Epilepsy is one of the most common neurological diseases affecting about 1% of the United States population.^1^ Epilepsy impacts all ages, races, social classes, and geographical locations.^2^ Uncontrolled seizures remain a significant problem in one third of treated patients.^3^, ^4^ Poor responses to ASMs persist despite the abundant availability of antiseizure medications (ASMs).^3^, ^4^, ^5^, ^6^, ^7^, ^8^ In drug‐resistant patients, the burden of epilepsy is further compounded. Denton et al.^9^ reported that up to 32% of patients with epilepsy become drug resistant adding to the clinical and economic burden of the condition.^10^

Moreover, epilepsy is not a standalone condition but a symptom of systemic dysfunction^11^ with comorbidities exacerbating the condition.^11^, ^12^, ^13^, ^14^ The latter authors reported the incremental cost for patients with epilepsy and mental health disorders increased the overall cost by 62%.

The purpose of the present study was to provide additional real‐world evidence on ASM prescribing patterns and related outcomes in patients diagnosed with focal epilepsy. Study focus was placed on patients who exhibited poor treatment response, aiming to identify related risk characteristics. Also, investigated were the associations of mental comorbidities, or the use of the following rescue medications: diazepam, lorazepam, midazolam, or alprazolam on treatment outcomes. The study utilized deidentified claims data with claims‐based definitions using specified International Classification of Diseases (ICD) codes as previously reported.^3^, ^13^, ^15^ Smith et al.^16^ reported that the use of ICD‐10‐CM claims‐based definitions for epilepsy and seizure type was relatively accurate.

## 2 METHODS

This retrospective study utilized IBM Explorys Therapeutic Datasets Delivered. Database provides deidentified real‐world data regarding patients, provider prescribing patterns, disease progression, procedures, and the overall delivery of care in the United States.

Identified patients were ≥18 years of age with a documented diagnosis of focal onset epilepsy between January 1, 2014 and December 31, 2019 (as defined in the 2017 ILAE Classification of Seizures). Patients were then selected if having ≥2 claims of any of the following diagnostic codes: ICD‐9 code 345.40 corresponding with ICD‐10 code G40.201 and G40.209; 345.41 corresponding with G40.211 and G40.219; 345.50 corresponding with G40.001, G40.009, G40.101, or G40.109; 345.51 corresponding with G40.011, G40.019, G40.111, or G40.119; 345.70 corresponding with G40.101 or G40.109; or 345.71 corresponding with G40.111, G40.119. The documented date of the second ICD diagnosis was defined as the study index diagnosis date.

Further, patients must have had ≥6 months of baseline period before study index diagnosis date and have received, in postindex diagnosis, one or in combination of any of the 10 selected ASMs indicated for monotherapy in focal epilepsy (Appendix A). An additional inclusion requirement was that patients must have had ≥1 year of continuous enrollment from index diagnosis date. This requirement was found restrictive and subsequently dropped because most patients were already on ASM treatment within the 6‐month baseline study period. Records were extracted for patients meeting the study inclusion criteria and had ASM‐related claim(s) identified by National Drug Codes (NDC). Follow‐up commenced on the index diagnosis date and continued until the patient exited the health plan or censored at the last date of data availability (December 31, 2019). Potential confounders and/or covariates included Quan Charlson Comorbidity Index (CCI^17^, ^18^), age, gender, and acute use of rescue medications either in baseline or postindex diagnosis. Also considered were patients with mental health conditions identified by ICD codes^13^ along with other commonly observed comorbidities.^11^

### 2.1 ASM‐related outcomes

The following mutually exclusive treatment outcomes were considered

1. Failure of therapy was defined as any changes in treatment postindex ASM regimen. Changes in doses and/or discontinuation of therapy were not counted as failure. Therapy failure was documented regardless of whether it was due to ineffectiveness or to adverse events/intolerance.
2. Patients were censored if they did not fail within ≤ year of follow‐up based on the absence of ASM‐related claims.
3. Patients who persisted on their index treatment for ≥365 days and did not experience therapy failure were deemed to have successful treatment response.
4. Patients who did not fail treatment and remained in their health insurance plan, without incurring any ASM prescription‐related claims for ≥365 days postindex ASM are considered as being seizure‐free.
5. Among patients who failed the first ASM regimen treatment, a second treatment failure was found when any change occurred for the second ASM regimen within the 1‐month titration period.
6. Patients with documented second treatment failures are deemed to be drug resistant.

### 2.2 Statistical analyses

Tabulated descriptive statistics are found below. At baseline, comorbid conditions were summarized using Quan CCI and the top four disease diagnoses related visits. Also counted were the use of rescue medications in baseline and postindex ASM.

Cox regression models estimated the hazard ratio and its confidence interval for treatment failure in patients initiated on any one of the nine ASMs listed in Appendix A, relative to those treated with levetiracetam as the most common first ASM. A similar approach was followed to quantify response failure among patients who failed their index ASM regimen. Models controlled for one or more of the baseline and demographic characteristics as potential confounders and covariates using forward‐stepwise selection and optimized Akaike Information Criterion (AIC). AIC is a mathematical method used to determine the best fit for the data.^19^ Standard Kaplan‐Meier (KM) curves were constructed to record the length of time from study ASM index date until failure, comparing the top five most frequently used ASMs, relative to time until treatment failure in patients treated with levetiracetam. Multivariable logistic regression with backward stepwise selection was performed to examine associations of ASM treatment outcomes with the use of rescue drugs in various care settings.

## 3 RESULTS

**FIGURE 1:**
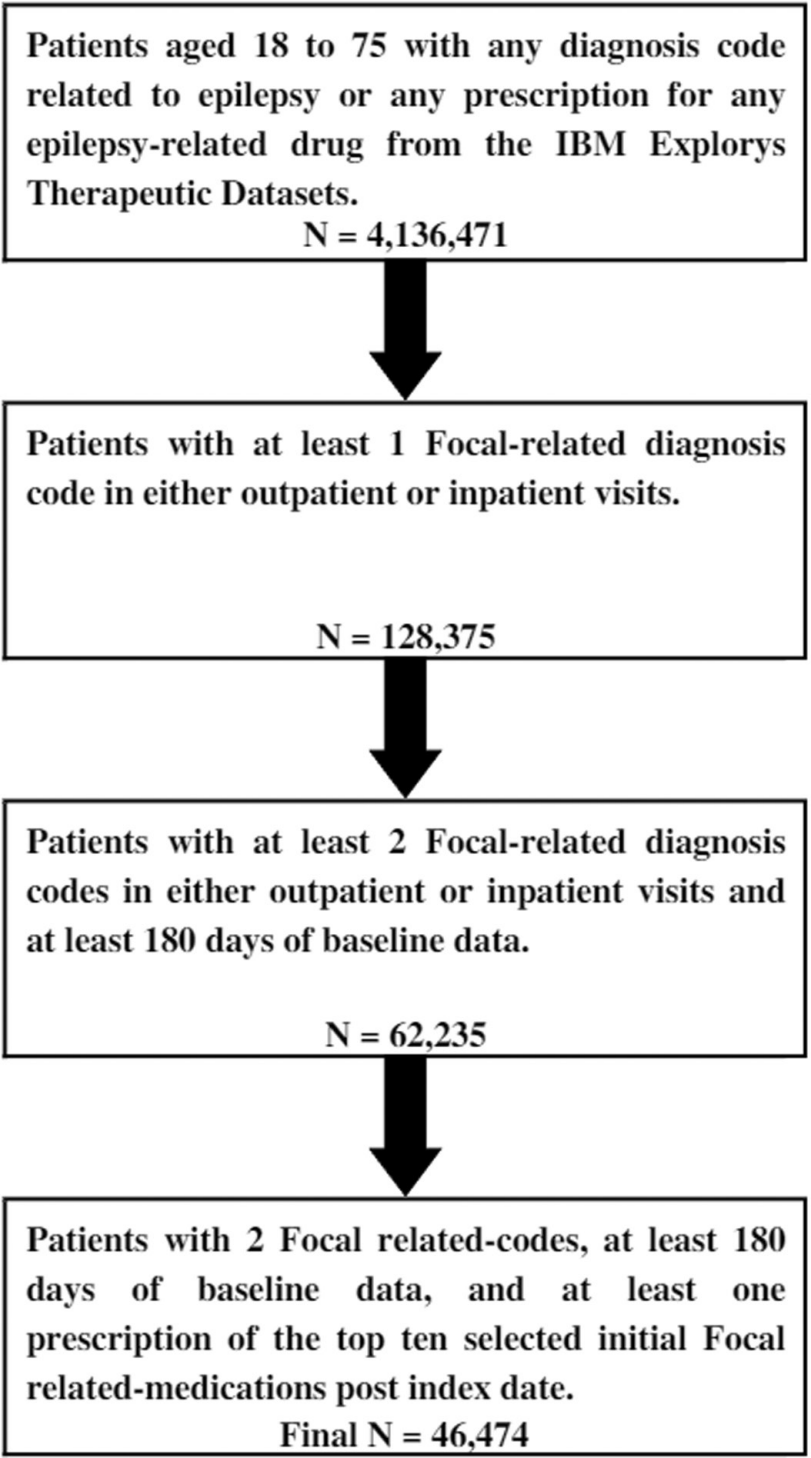
Selection process for the final study cohort.

Patients' identification process is depicted in Figure 1. The final study cohort consisted of 46 474 patients who met all inclusion criteria.

### 3.1 Descriptive statistics

Table 1 details baseline demographics and other characteristics of the study cohort. The mean age (at the end of the baseline period) was 47.23 (SD: 16.94) and 44.25% were male, with mean CCI of 0.97 (SD: 1.82). Comorbid mental conditions were present in 17 594 patients (37.86%), with the most prevalent condition being depressive episodes (7.60%). At baseline, the most common visits were for hypertension (21.67%), general outpatient, headache, and syncope, with relative frequencies of 14.56%, 11.58%, and 8.40%, respectively.

**TABLE 1:** Baseline period characteristics of the study cohort (*N* = 46 474).

Variables	Statistics
Demographics at index date	
Age, Mean (SD); Range (min, max)	47.23 (16.94); (18, 101)
Gender (Male), *N* (%)	20 567 (44.25)
Baseline comorbidity	
CCI^a^, Mean (SD); Range (min, max)	0.97 (1.82); (0, 16)
Any mental health condition, *N* (%)^b^	17 594 (37.86)
Depressive episode	3530 (7.60)
Generalized anxiety disorder	2267 (4.88)
Recurrent depressive disorder, moderate	1407 (3.03)
Bipolar affective disorder, unspecified	631 (1.36)
Recurrent depressive disorder, severe	598 (1.29)
Baseline outpatient visits by four primary DX encountered	*Mean (SD); Range (min, max)*
Syncope	0.85 (5.08); (0, 175)
Hypertension	2.45 (8.87); (0, 348)
Headache	0.73 (3.77); (0, 124)
General medical visit	0.78 (2.69); (0, 69)
Proportions with any baseline outpatient visits by DX	*N* (%)
Hypertension	10 073 (21.67)
General visits	6765 (14.56)
Headache	5382 (11.58)
Syncope	3906 (8.40)

Abbreviation: DX, diagnosis.

^a^ Quan Charlson Comorbidity Index.

^b^ Selected conditions denote some of the most commonly encountered codes.

Levetiracetam was the most widely used ASM at initiation (index) with 17 630 patients (37.94%), followed by lamotrigine (21.34%), carbamazepine (8.81%), topiramate (7.25%), oxcarbazepine (7.20%), lacosamide (7.02%), valproate (5.49%), phenytoin (4.17%), primidone (0.56%), and perampanel (0.23%). At baseline, 87.14% of the patients were already on with their index ASM regimen. Also, at baseline, 27.38% of patients were on 1 ≥ ASM different than that found in the index (titration) ASM regimen. Only 7.0% of the patients were not on an ASM prior to the index regimen. Rescue medication use was documented for 4.19% of the patients (1948).

Table 2 details patients' outcomes associated with the index ASM regimens, followed by the second ASM regimens in patients who failed their first regimen. Among the 46 474 patients, 16 083 patients, 34.61% successfully persisted on their index regimen for the duration of first‐year follow‐up, 2746 patients (5.91%) were seizure‐free, 12 868 patients (27.69%) failed treatment, while the remaining 31.80% patients were censored. On average, patients persisted on their first‐year regimen for 218.36 days (SD: 142.22) received 7.76 ASM prescription claims (SD: 7.49) with total supply of days of 329.77 (SD: 264.05). Patients who failed their second treatment regimen numbered 6335 (49.23% of the 12 868 patients who failed their first regimen). Percentages of patients deemed to have successful second regimen treatment, seizure‐free, or censored were 21.32%, 3.65%, and 25.80%, respectively. On average, patients persisted on their second ASM regimen for 165.41 (137.04) days received a total of 8.29 claims (SD: 8.62), with an average total days' supply of 329.89 (SD: 307.22).

**TABLE 2:** Treatment outcomes in study cohort postindex and second ASM treatment regimens.

Variables	Statistics
Outcomes associated with index regimen	
Patients' total	46 474 (100)
Success (by the end of the first year)	16 083 (34.61)
Seizure‐free	2746 (5.91)
Failed, *N* (%)	12 868 (27.69)
Censored^a^, *N* (%)	14 777 (31.80)
Other outcome indicators in first year postindex ASM	*Mean (SD); Range (min, max)*
Time to fail	218.36 (142.22); (0, 365)
Number of prescriptions	7.76 (7.49); (1, 97)
Days of supply	329.77 (264.05); (1, 2648)
Second treatment outcome (in failed patients, *N*, %)	12 868 (100)
Success on second ASM regimen	2743 (21.32)
Failed response	6335 (49.23)
Seizure‐free on second regimen	470 (3.65)
Censored on second regimen^b^	3320 (25.80)
Other outcome indicators in second regimen treated patients (in failed patients)	*Mean (SD); Range (min, max)*
Time to fail	165.41 (137.04); (0, 365)
Number of prescriptions	8.29 (8.62); (1, 96)
Days of supply	329.89 (307.22); (1, 2820)

Abbreviation: ASM, antiseizure medication.

^a^ Censored denotes patients persisted on first regimen but did not have 1 year of follow‐up after first index ASM regimen.

^b^ Censored denotes patients persisted on second regimen but did not have 1 year of follow‐up after second ASM regimen.

**FIGURE 2:**
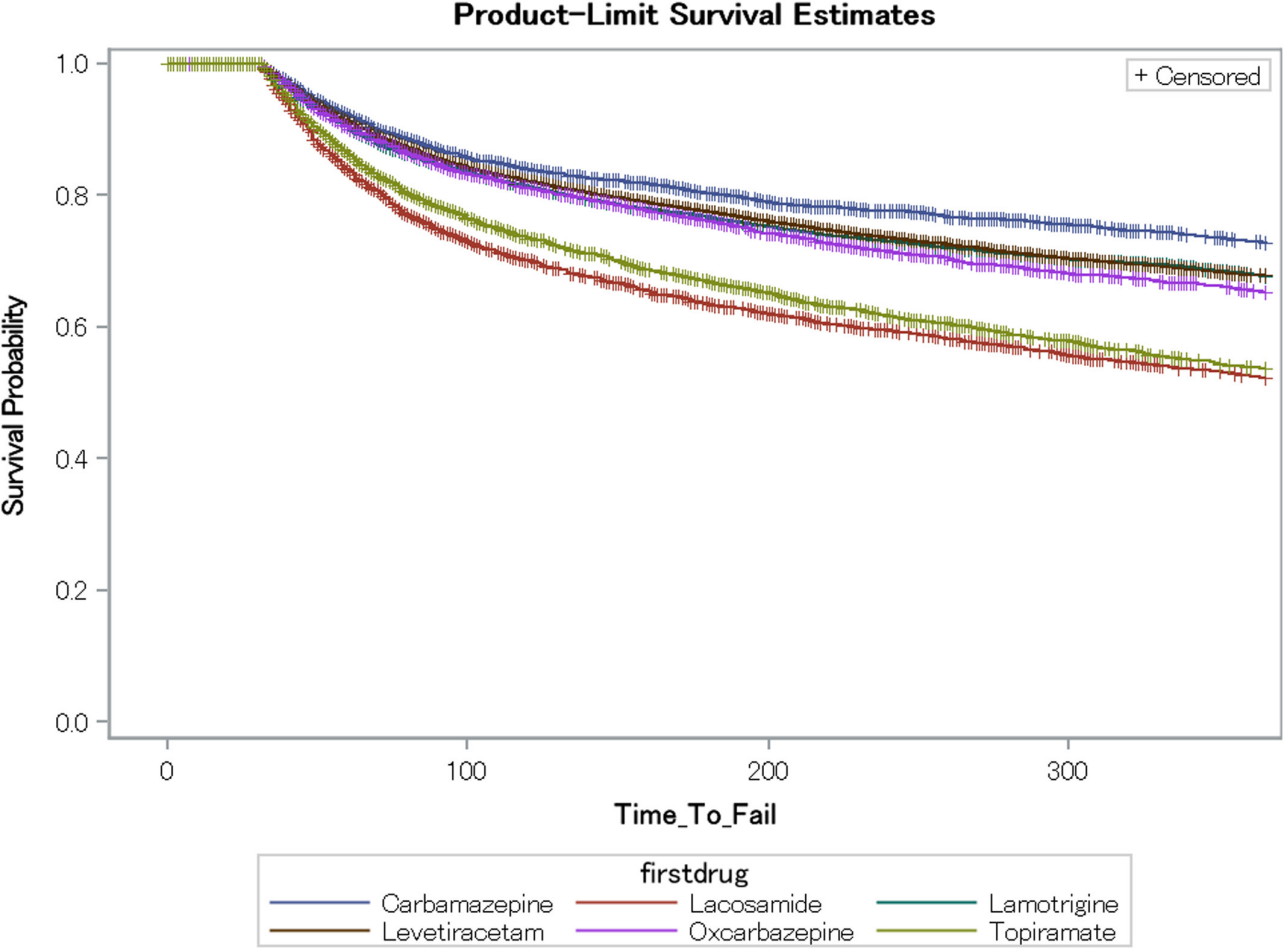
Kaplan‐Meier curves for time to fail of the top six first antiseizure medication index regimens.

Standard KM curves were constructed for the top six index ASM regimens (Figure 2). Lacosamide had the shortest time to fail, albeit all six ASMs had relatively comparable curves.

Cox regression models on risk of failing index ASM regimen are presented in Table 3. Relative to patients who started with levetiracetam, patients on lamotrigine or carbamazepine, had significantly lower risk (hazard) of treatment failure. Conversely, patients initiated on perampanel, phenytoin, valproate, topiramate, or lacosamide experienced significantly higher risk of failing treatment. Baseline use of an index ASM or rescue medications reduced the risk of failing treatment, while baseline use of ASMs other than those found in the index regimen increased the risk of failing. Older and male patients had lower risk of failing treatment relative to younger patients or females. Patients with any of the following baseline risk factors: higher CCI, any mental illness as comorbidity, bipolar affective disorder, recurrent moderate depressive disorder, headache, or neoplasty all had significantly greater risk of treatment failure.

**TABLE 3:** Cox regression models on risk of index ASM treatment failure (*N* = 46 474).

Parameter	Hazard ratio	HR lower bound	HR upper bound	*P*‐value
Index ASM				
Levetiracetam (reference)	1.000	‐	‐	‐
Carbamazepine	0.922	0.858	0.991	0.027
Lamotrigine	0.928	0.883	0.975	0.003
Oxcarbazepine	0.946	0.881	1.016	0.125
Perampanel	2.076	1.620	2.660	<0.0001
Phenytoin	1.116	1.018	1.224	0.019
Primidone	1.055	0.841	1.324	0.643
Valproate	1.084	1.004	1.169	0.038
Topiramate	1.225	1.148	1.306	<0.0001
Lacosamide	1.204	1.130	1.283	<0.0001
Age at index	0.996	0.995	0.997	<0.0001
Male	0.901	0.869	0.934	<0.0001
CCI	1.025	1.014	1.035	<0.0001
Rescue drug in baseline	0.634	0.590	0.683	<0.0001
Use of first index ASM in baseline	0.920	0.876	0.966	0.001
Other than index ASM in baseline	4.755	4.582	4.935	<0.0001
Any mental health condition in baseline^a^	1.173	1.129	1.218	<0.0001
Bipolar affective disorder, unspecified in baseline	1.196	1.061	1.349	0.003
Generalized anxiety disorder	1.069	0.993	1.150	0.076
Other specified mental disorders due to brain damage and dysfunction in baseline	0.787	0.602	1.029	0.080
Recurrent depressive disorder, moderate in baseline	1.149	1.053	1.254	0.002
Baseline period headache visit	1.004	1.000	1.008	0.028
Baseline period neoplasty visit	1.006	1.004	1.008	<0.0001
Baseline period general medication visit	0.995	0.988	1.001	0.091

Abbreviations: ASM, antiseizure medication; CCI, Charlson Comorbidity Index.

^a^ This category includes all mental conditions. Rows below reflect specific mental conditions as stated.

Results of Cox regression models on risk of failing a second ASM regimen in patients who failed their first regimen are presented in Table 4. Baseline use of any of the index ASMs, having other than index (titration) ASM regimen, having been diagnosed with depressive episode, or generalized anxiety disorder all resulted in higher risk for failing second ASM regimen.

**TABLE 4:** Cox regression models on risk of second ASM regimen treatment failure in patients who failed index ASM.

Parameter	Hazard ratio	HR lower bound	HR upper bound	*P*‐value
Index ASM				
Levetiracetam (reference)	1.000	‐	‐	‐
Carbamazepine	1.023	0.924	1.132	0.661
Lamotrigine	1.050	0.979	1.125	0.170
Oxcarbazepine	1.043	0.942	1.155	0.415
Perampanel	1.126	0.793	1.599	0.506
Phenytoin	1.135	0.999	1.289	0.052
Primidone	1.277	0.940	1.734	0.118
Valproate	0.975	0.875	1.087	0.651
Topiramate	1.065	0.974	1.165	0.165
Lacosamide	0.983	0.897	1.076	0.706
Age at index	1.001	1.000	1.003	0.060
Use of index ASM in baseline	1.123	1.045	1.206	0.002
Other than index ASM regimen use in baseline	1.218	1.158	1.281	<0.0001
Depressive episode	1.125	1.037	1.220	0.005
Generalized anxiety disorder	1.111	1.007	1.227	0.036
Recurrent depressive disorder, moderate	1.117	0.992	1.257	0.067
Baseline headache visit	1.004	0.999	1.010	0.132

Abbreviation: ASM, antiseizure medication.

### 3.2 Impact of rescue medication use post‐ASM treatment

We calculated the odds ratios and confidence intervals for variables found to have associations with the need (risk) for rescue medication utilizations in either outpatient setting, emergency department (ED), or in urgent care setting. Age at index, or male gender, afforded patient protection from having to use rescue medications in an outpatient setting. Conversely, use of rescue medications at baseline, use of either lamotrigine, topiramate, or lacosamide as the index ASMs, having depressive episodes, having other than index ASM at baseline, having mental comorbidity, or having either headache or neoplasty at baseline were all associated with higher needs for rescue medications administered in outpatient settings. Similarly, older age at index or having an index regimen at baseline afforded patients the protection from having the need for rescue medications administered in ED. Conversely, higher CCI, having other than index ASM at baseline, having any mental condition, having bipolar affective disorder with severe depression, or baseline visits with either headache or neoplasty were all associated with higher needs for rescue medication use in ED. The use of rescue medications at baseline was the only variable found associated with the need for rescue medication use postindex ASM treatment in urgent care settings. Tabulated results can be found in the Supporting Information.

## 4 DISCUSSION

This retrospective study aimed to provide real‐world evidence on treatment patterns and related outcomes for the most frequently encountered ASMs in the study cohort. Most patients on 1 ≥ ASM(s) prior to having a confirmed focal epilepsy diagnosis per study protocol. More than one third of the patients were treated first with levetiracetam. One third of the study cohort successfully persisted on their index regimen for the first year posttreatment. Over one fourth of the study cohort failed their index ASM regimen. Also, almost half of those treated with a second ASM regimen, having failed their first regimen, subsequently failed treatment.

Patients initiated on either lamotrigine or carbamazepine as their index ASM experienced a lower risk of treatment failure compared to the levetiracetam‐treated group. The use of index ASM, or rescue medications at baseline, bestowed protection from the risk of treatment failure. Older or male patients experienced a lower risk of failing treatment, while patients with higher comorbidities were more likely to fail. Comorbid mental illnesses increase the risk of treatment failure. In addition, having baseline headache or neoplasty increases the risk of treatment failure. The latter findings corroborate an earlier report on the burden of comorbidities, such as depression and migraine, on seizure outcomes.^12^

Our findings on the negative impact of mental comorbidities on patients' treatment corroborated the results reported by Petrilla et al.^13^ Moreover, in the present study, both depression and anxiety negatively impacted ASM treatment outcome. This is noteworthy since both conditions were the most reported comorbidities in the aforementioned study.

Two findings regarding ASM treatment outcomes are worthy of discussion. First, the choice of initial ASM regimen seemed to impact the outcome. Findings lend credence to a recent report on the failure to respond to first ASM as a significant risk factor for treatment failure.^9^ Also, notwithstanding whether baseline rescue medications were to manage mental comorbidities, its use was associated with better treatment outcomes.

Devinsky et al. advocated the use of big data to influence treatment choice in epilepsy.^20^ These authors reasoned that clinicians' ability to identify ASM regimens to ensure the best possible patient outcomes remains a challenge since neither clinical trials nor existing epilepsy treatment guidelines provide recommendations on key aspects of care such as treatment for drug‐resistant epilepsy. Reported results herein may prove useful in further elucidating the complex nature of pharmacological treatment for focal epilepsy, and the intertwined relationships between treatment options and patients' independent risk characteristics.

### 4.1 Study limitations

Since seizure as the primary symptom of epilepsy is not captured with claims data, treatment changes have been used as a proxy for seizure control and/or patient outcome status.^20^ The logic here is that the need to change an existing ASM treatment reflects suboptimal effect and/or its lack of tolerability. However, ascertaining the rationale for ASM treatment changes due to adverse events could only be done by reviewing clinician notations in patients' charts. Furthermore, it was not possible to invoke the usual paradigm of pharmacoepidemiologic studies by first selecting patients based on indication‐designated ICD diagnostic codes. Only 7% of the study cohort did not have ASM claims prior to the ICD‐related diagnoses.

## 5 CONCLUSIONS

Overall, this study's findings help elucidate the complex nature of the pharmacological treatment of drug‐resistant focal epilepsy and the need for additional research to improve the potential to achieve an impactful benefit of early and appropriate treatment on patient outcomes.

## AUTHOR CONTRIBUTIONS

Hind T. Hatoum was involved in the study design, analyses of data, and writing of the manuscript. Steve Arcona was involved in the study design, data interpretation, and writing of the manuscript. Jianbin Mao was involved in the interpretation of data and manuscript writing. Surrey Walton was involved in data analyses and manuscript writing.

## CONFLICT OF INTEREST STATEMENT

Dr. Hind T. Hatoum received financial support through contractual agreement between Hind T. Hatoum & Company and Cerevel Therapeutics, LLC. Both Drs Steve Arcona and Jianbin Mao are paid salaried employees of Cerevel Therapeutics, LLC. Dr. Surrey Walton is the Managing Principal for Outcome City Consulting Company that acted as a subcontractor to Hind T. Hatoum & Company for data analyses.

## ETHICS STATEMENT

This article does not contain any studies involving human participants. Dataset used deidentified real‐world patient information. The present data cannot be shared due to legal restrictions. Hind T. Hatoum contributed to all aspects of this manuscript. Surrey Walton provided the data analyses and contributed to the writing of the manuscript. Both Steve Arcona and Jianbin Mao provided input into the study analyses and participated in the manuscript writing and editing.

## Supporting information


Data S1.

## APPENDIX A

### A.1 ASMs indicated alone or in combination with other ASMs

**Table:**

ASMs indicated as monotherapy
Carbamazepine
Lamotrigine
Oxcarbazepine
Perampanel
Phenytoin
Primidone
Topiramate
Valproate (Divalproex + valproic acid + valproate)
Lacosamide
Levetiracetam
ASMs indicated as cotherapy
Brivaracetam
Clonazepam
Clorazepate
Eslicarbazepine
Ezogabine
Felbamate
Gabapentin
Phenobarbital
Pregabalin
Tiagabine
Zonisamide
Rescue medications considered
Diazepam
Lorazepam
Midazolam
Alprazolam
