# Supplementary material for: Real‐world antiseizure medication treatment outcomes in drug‐resistant focal epilepsy patients
Source: Epilepsia Open. 2023 Oct 31;8(4):1556–65. doi: 10.1002/epi4.12845 (PMC10690664; doi:10.1002/epi4.12845)
Supplement: Supplementary file 1 — Data S1. [file EPI4-8-1556-s001.docx]

**Hatoum et al. Supplement**

**Impact of Rescue Medications Use on ASM treatment Outcome.**

## Table 1. Impact of Independent Variables on Risk/ Protection of Rescue Medications Administered in Outpatient Clinics

| **Variable** | **Odds**  **Ratio** | **OR Lower Bound** | **OR Upper**  **Bound** | **P-**  **Value** |
| --- | --- | --- | --- | --- |
| Index ASM |  |  |  |  |
| Levetiracetam (reference) | 1.000 |  |  |  |
| Lamotrigine first* | 1.134 | 1.021 | 1.26 | <.0001 |
| Valproate First | 1.166 | 0.977 | 1.393 | 0.0188 |
| Topiramate First | 1.177 | 1.01 | 1.371 | 0.0892 |
| Lacosamide First | 1.345 | 1.161 | 1.558 | 0.0367 |
| Patient age at index | 0.985 | 0.983 | 0.988 | <.0001 |
| Male | 0.847 | 0.777 | 0.922 | <.0001 |
| CCI | 1.101 | 1.079 | 1.125 | 0.0001 |
| Rescue drug used in baseline | 5.618 | 4.933 | 6.399 | <.0001 |
| Other that index ASM regimen in baseline | 1.129 | 1.022 | 1.246 | <.0001 |
| Any mental health condition | 1.425 | 1.31 | 1.551 | 0.0164 |
| Moderate depressive episode | 0.618 | 0.374 | 1.02 | <.0001 |
| Baseline outpatient visits for headache | 1.008 | 1 | 1.017 | 0.0599 |
| Baseline outpatient visits for neoplasty | N1.008 | 1.004 | 1.011 | 0.0478 |

## *First ASM encountered

## Table 2. Impact of Independent Variables on Risk/ Protection of Rescue Medications Administered in Emergency Departments (ED)

| **Variable** | **Odds**  **Ratio** | **OR Lower Bound** | **OR Upper Bound** | **P-**  **Value** |
| --- | --- | --- | --- | --- |
| Index ASM |  |  |  |  |
| Levetiracetam (reference) | 1.000 |  |  |  |
| Patient age at index | 0.982 | 0.977 | 0.988 | <.0001 |
| CCI | 1.068 | 1.022 | 1.117 | 0.0038 |
| Rescue drug used in baseline | 2.902 | 2.202 | 3.824 | <.0001 |
| Use of index ASM in baseline | 0.737 | 0.587 | 0.927 | 0.0090 |
| Other than index ASM regimen used in baseline | 1.323 | 1.083 | 1.617 | 0.0061 |
| Any mental health condition in baseline | 1.404 | 1.174 | 1.678 | 0.0002 |
| Bipolar affective disorder, current episode severe depression without psychotic symptoms in baseline | 4.401 | 1.744 | 11.111 | 0.0017 |
| Baseline outpatient visits for headache | 1.021 | 1.009 | 1.034 | 0.0005 |
| Baseline outpatient visits for neoplasty | 1.009 | 1.003 | 1.015 | 0.0032 |

## Table 3. Impact of Independent Variables on Risk/Protection of Rescue Medications Administered in Urgent Care Settings

| **Variable** | **Odds Ratio** | **OR Lower Bound** | **OR Upper Bound** | **P-**  **Value** |
| --- | --- | --- | --- | --- |
| Rescue drug used in baseline | 6.66 | 2.952 | 15.026 | <.0001 |
| Depressive episode in baseline | 2.03 | 0.8 | 5.152 | 0.1364 |
| Bipolar affective disorder, unspecified in baseline | 3.201 | 0.739 | 13.871 | 0.1199 |
| Other specified mental disorders due to brain damage  and dysfunction in baseline | 6.663 | 0.882 | 50.332 | 0.0660 |
| Baseline outpatient visits for headache | 1.028 | 0.998 | 1.059 | 0.0690 |
